# A nomogram for individualized prediction of new-onset postoperative atrial fibrillation in acute type A aortic dissection patients: a retrospective study

**DOI:** 10.3389/fcvm.2024.1429680

**Published:** 2024-08-21

**Authors:** Zhihao Yang, Chunxiao Liu, Chao Fu, Xin Zhao

**Affiliations:** ^1^ Department of Cardiovascular Surgery, Qilu Hospital of Shandong University, Jinan, China; ^2^ Institute of Thoracoscopy in Cardiac Surgery, Shandong University, Jinan, China

**Keywords:** new-onset postoperative atrial fibrillation, acute type A aortic dissection, Sun's surgery, risk factors, prediction model

## Abstract

**Objective:**

The objective of this study is to explore the risk factors associated with new-onset postoperative atrial fibrillation (POAF) following Sun's surgery(total arch replacement using a tetrafurcate graft with stented elephant trunk implantation) for acute type A aortic dissection(AAAD) and to develop a predictive model for assessing the likelihood of new-onset POAF in patients undergoing Sun's surgery for AAAD.

**Methods:**

We reviewed the clinical parameters of patients diagnosed with AAAD who underwent Sun's surgery at Qilu Hospital between December 1, 2017 and December 31, 2022. The data was analyzed through univariable and multivariable logistic regression analysis. Variance inflation factor was used to investigate for variable collinearity. A nomogram for predicting new-onset POAF was developed and verified by bootstrap resampling. In addition, the calibration of our model was evaluated by the calibration curve and Hosmer-Lemeshow test. Furthermore, the clinical utility of our model was evaluated using the net benefit curve.

**Results:**

This study focused on a cohort of 242 patients with AAAD, among whom 42 experienced new-onset POAF, indicating an incidence rate of 17.36%. Age, left atrial diameter (LA), right atrial diameter (RA), preoperative red blood cells (RBC), and previous acute coronary syndrome (preACS) emerged as independent influences on new-onset POAF following Sun's surgery, as identified by univariable and multivariable logistic regression analysis. Collinearity analysis with demonstrated no collinearity among the variables. A user-friendly prediction nomogram for new onset POAF following Sun's surgery was formulated. The model demonstrated commendable diagnostic accuracy with an area under the curve (AUC) of 0.7852. Validation of the model through bootstrapping (1,000 repetitions) yielded an AUC of 0.8080 (95% CI: 0.8056–0.8104). affirming its robustness. Additionally, the model exhibited favorable fit, calibration, and positive net benefits in decision curve analysis.

**Conclusions:**

Drawing upon these findings, we have developed a predictive model for the occurrence of new-onset POAF. These results suggest the potential efficacy of this prediction model for identifying patients at risk of developing POAF. The visualization of this model empowers healthcare professionals to conveniently and promptly assess the risk of AF in patients, thereby facilitating the timely intervention implementation.

## Introduction

Atrial fibrillation (AF), an arrhythmia, frequently occurs following cardiac surgery (1). The incidence of postoperative atrial fibrillation (POAF) in individuals with cardiac surgery ranges from 10% to 52.7% (2, 3). The rate of POAF depends on the definition of the condition, monitoring methods used, and the level of risk in the study population (4). Acute type A aortic dissection (AAAD) presents a significant threat to cardiovascular health, marked by a spectrum of severe complications including hypotension, shock, pericardial effusion and tamponade, periaortic hematoma, renal insufficiency, mesenteric malperfusion, and brain injury (5). Timely surgical intervention constitutes the primary treatment strategy for AAAD. The frozen elephant trunk and total arch replacement, colloquially referred to as the Sun's surgery, has emerged as the standard surgical approach for AAAD in China and has been gained recognition globally towing to its favorable outcomes in both short-term and long-term perspectives (6, 7). Although advancements in surgical techniques and postoperative care have improved outcomes and reduced rates of mortality and serious complication (2), the incidence of AF following surgery remains unabated, which may attribute to the increasing age of the surgical population (8, 9). Postoperative AF cardiac patients typically manifests within the second to fourth day after surgery, often resolving spontaneously and being of a transient nature (10). However, it can led to early complications, particularly in the elderly or those with pre-existing conditions, such as congestive heart failures, hemodynamic instability, acute kidney injury, acute heart failure, and stroke (11–14). Research has highlighted the association of postoperative AF with increased mortality, heightened rates of stroke and heart failure, prolonged hospitalization, and increased hospital costs (15, 16).

Efforts to prevent and manage POAF following AAAD have proven challenging and ineffective, with no significant change in incidence over the past several decades (17, 18). Identifying factors that influence POAF is of significant importance for effective prevention and treatment. Therefore, it is crucial to anticipate the occurrence of new-onset AF after Sun's surgery. However, there is a limited focus on the specific risk factors for new-onset POAF specifically for AAAD (2). Therefore, this retrospective study aimed to analyze clinical data of patients undergoing Sun's surgery for AAAD to validate previously identified factors and explore new ones influencing new-onset POAF in AAAD. Potential risk factors for predicting new-onset POAF in AAAD patients were identified, and a simple, visually intuitive, and clinically applicable risk prediction model was developed in the form of a line chart was developed to facilitate early and effective identification of high-risk individuals for new-onset POAF in AAAD patients, enabling timely interventions. This risk prediction model offers a theoretical foundation for tailoring treatment regimens to decrease the incidence of new-onset POAF in patients with AAAD following Sun's procedure. Furthermore, the metrics depicted in this model are derived from easily accessible clinical data, ensuring their simplicity and ease of use.

According to the Society of Thoracic Surgeons National database (located at http://sts.org), POAF in our research was classified based on the presence of AF persisting for five minutes or more on two or more consecutive days of electrocardiogram monitoring, or the occurrence of atrial flutter necessitating intervention (utilizing medications such as β-blockers, calcium channel blockers, amiodarone, etc.) (8). Upon being diagnosed with AF, patients received either cardioversion or one or more medical treatments, or both. For cases of persistent or recurrent atrial fibrillation, anticoagulation with warfarin was initiated.

## Methods

### Patients and data collection

**Figure 1:**
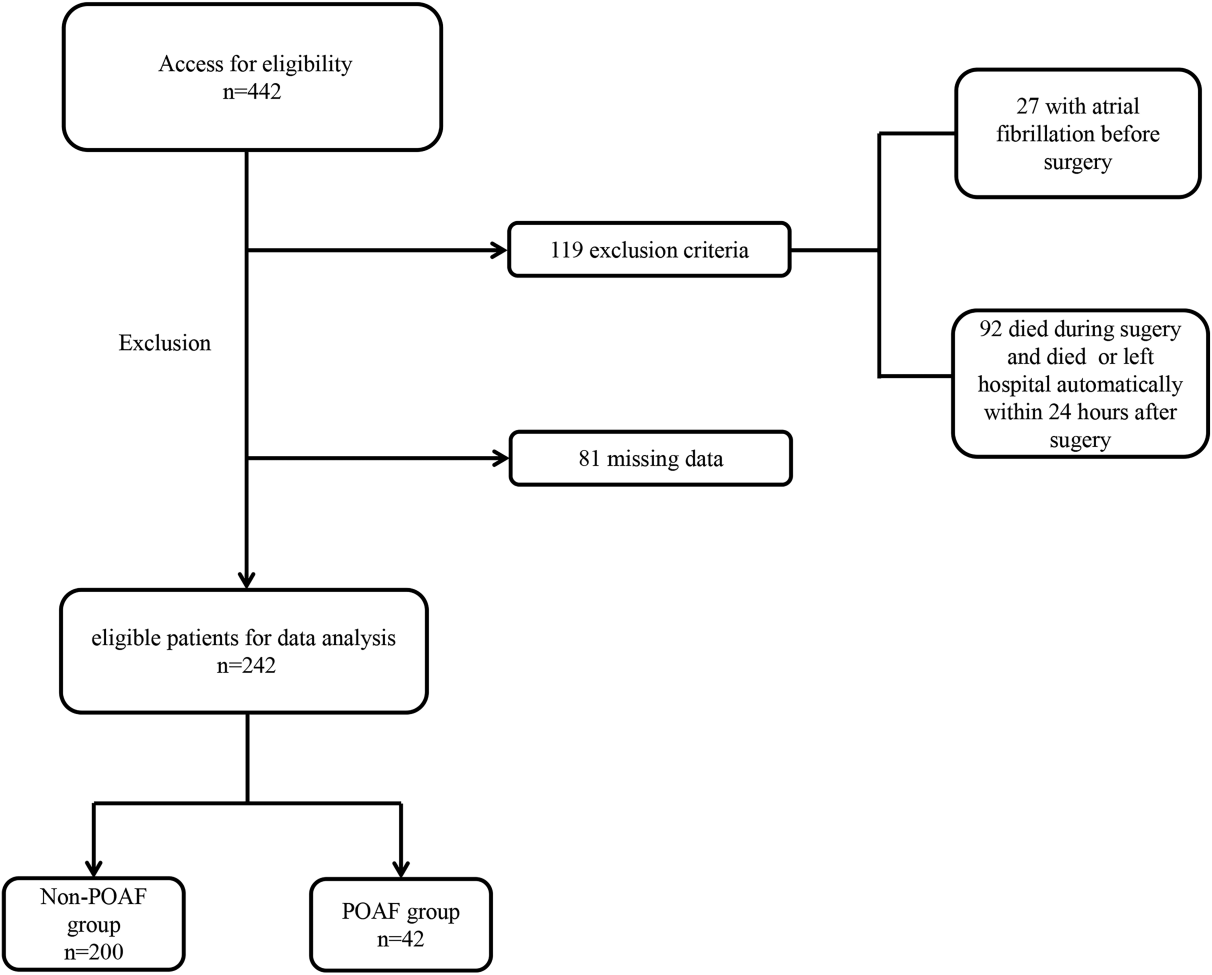
Flow chart for selection of the study population. POAF, postoperative atrial fibrillation.

Between December 1, 2017, and December 31, 2022, we retrospectively reviewed 442 patients diagnosed with AAAD who underwent Sun's surgery at Qilu Hospital, Shandong University. Exclusion criteria (Figure 1) were (1) patients under 16 years old, (2) patients with pre-existing AF, (3) patients who passed away within 24 h after surgery, (4) patients who left hospital automatically within 24 h after surgery, (5) cases with missing data. Ultimately, 242 patients were included in the analytic cohort. A standardized survey form was designed, and relevant information was from the electronic patient record system, including demographic information (age, gender), lifestyle behaviors (history of smoking and history of drinking), physical examination (height, weight, etc.), medical history, preoperative 24-hour laboratory tests, and echocardiography, etc. The primary outcome measure of this study was the presence of new-onset AF after operation.

### Statistical analysis

Statistical analysis was conducted using SPSS 26.0 software. The Shapiro—Wilk procedure was employed to assess the normality of the measurements. Data were presented as mean ± standard deviation (normal distribution) and median (P25,P75) (non normal distribution). Comparisons between groups were conducted using the t-test and rank-sum test, respectively. Categorical variables were reported in terms of whole numbers and proportions, with differences between groups being assessed by using the *χ*^2^ test. Univariate analysis was applied to all clinical variables, and factors with predictive potential were identified (*P *< 0.05). Subsequently, univariable logistic regression analysis was conducted. A multivariable logistic regression analysis was carried out, considering variables with *P* values less than 0.05 and incorporating professional expertise, to determine the risk factors. Variance inflation factors (VIFs) was calculated to assess the collinearity assumption, with VIF less than 5 considered to indicate no significant collinearity. A nomogram predicting the probability of new-onset POAF rates after Sun's surgery was constructed using the rms package of R, version 4.2.2 (http://www.r-project.org/). The model's discrimination capability was assessed using the area under the receiver operating characteristic curve. The accuracy of our model was further verified by bootstrap validation using computer resampling for 1,000 repetitions of simple random sampling with replacement. The calibration of our model was evaluated by the calibration curve and Hosmer–Lemeshow test (HL test). Assessment of the clinical utility of our model was conducted using the net benefit curve, forllowing the methodology established by Vickers et al (19). A *P* value less than 0.05 was considered statistically significant.

## Results

### Demographic and clinical characteristics

The median age of patients was 51 years (IQR, 42–61 years), with females comprising 30.2% (73 of 242) of the patient population. Out of a total of 42 new-onset POAF patients, 32 (76.2%) had hypertension, and 2 (4.8%) had diabetes mellitus. 31.0% (14 of 42) of new-onset POAF patients had a history of previous acute coronary syndrome. New-onset POAF patients exhibited an average left atrial diameter size of 38.48 ± 7.91 mm, whereas Non-POAF patients had an average of 35.86 ± 6.02 mm. Additional patient characteristics are detailed in Table 1.

**Table 1:** Baseline characteristic of the 242 AAAD patients.

	Total (*n* = 242)	Non-POAF group (*n* = 200)	POAF group (*n* = 42)	*P* valve
Age (years)	51.00 (42.00, 61.00)	50.00 (42.00, 60.00)	56.00 (51.00, 66.00)	0.001
Weight (kg)	74.50 (65.00, 83.00)	74.00 (65.00, 82.12)	75.00 (63.25, 84.75)	0.554
Gender, female (%)	73 (30.2)	56 (28.0)	17 (40.5)	0.157
Smoking history (%)	93 (38.4)	76 (38.0)	17 (40.5)	0.9
Drinking history (%)	117 (48.3)	96 (48.0)	21 (50.0)	0.947
Medical history				
HT(%)	164 (67.8)	132 (66.0)	32 (76.2)	0.27
DM(%)	11 (4.5)	9 (4.5)	2 (4.8)	0.931
preACS(%)	27 (11.2)	14 (7.0)	13 (31.0)	<0.001
Preoperative test				
LA (mm)	36.31 ± 6.45	35.86 ± 6.02	38.48 ± 7.91	0.016
LV (mm)	45.00 (42.00, 50.00)	45.00 (42.00, 51.00)	46.00 (42.00, 50.00)	0.822
RA (mm)	44.00 (39.00, 49.00)	44.00 (39.00, 49.00)	45.00 (41.25, 53.00)	0.039
RV(mm)	23.00 (20.00, 25.00)	23.00 (20.00, 25.00)	22.00 (20.25, 24.00)	0.627
LVEF	0.61 (0.58, 0.65)	0.60 (0.58, 0.65)	0.61 (0.59, 0.65)	0.767
WBC (×10^9^ /L)	9.87 (7.68, 12.30)	10.02 (7.83, 12.58)	8.93 (6.94, 10.82)	0.048
RBC (×10^9^ /L)	4.01 (3.64, 4.40)	4.05 (3.70, 4.43)	3.92 (3.32, 4.26)	0.023
HGB (g/L)	124.00 (111.00, 135.00)	124.00 (111.75, 138.00)	122.50 (100.75, 134.00)	0.16
PLT (×10^9^ /L)	167.50 (136.25, 208.25)	170.00 (136.75, 209.25)	152.50 (132.50, 203.50)	0.219
PTINR	1.11 (1.05, 1.20)	1.11 (1.04, 1.19)	1.12 (1.07, 1.20)	0.149
CKMB (ng/ml)	1.85 (0.80, 6.27)	1.65 (0.80, 5.62)	2.70 (1.00, 8.85)	0.09
FIB (g/L)	3.26 (2.50, 4.26)	3.40 (2.55, 4.35)	2.80 (2.32, 3.62)	0.045
NLR	7.47 (4.81, 12.28)	7.38 (4.83, 12.14)	7.62 (4.69, 13.56)	0.597
Intraoperative variables				
Surgerytime (min)	478.72 ± 94.14	477.80 ± 97.16	483.10 ± 79.06	0.741
ECC (min)	217.50 (189.00, 252.00)	215.00 (184.00, 254.00)	219.50 (198.50, 244.00)	0.426
Aortic cross clamping time (min)	137.00 (117.00, 159.00)	136.00 (115.00, 159.00)	141.00 (128.00, 164.00)	0.188
Circulatory arrest time (min)	16.00 (19.00, 35.00)	26.00 (20.00, 34.00)	22.00 (17.00, 37.00)	0.574

HT, hypertension; DM, diabetes mellitus; preACS, previous acute coronary syndrome; LA, left atrial diameter; LV, Left ventricular diameter; RA, right atrial diameter; RV, right ventricular diameter; LVEF, left ventricular ejection fraction; WBC, preoperative white blood cells; RBC, preoperative red blood cells; HGB, preoperative hemoglobin; PLT, preoperative platelet count; PTINR, preoperative prothrombin time nternational normalized ratio; CKMB, preoperative creatine kinase isoenzymes; FIB, preoperative fibrinogen; NLR, preoperative neutrophil to lymphocyte ratio; ECC, extracorporeal circulation time.

### Selected factors for model

Combined with the results of the univariate analysis, seven factors with potential predictive ability (*P *< 0.05) were identified among all the factors and subjected to univariable logistic regression analysis. The findings are presented in Table 2. Subsequently, factors showing likely predictive capability (*P *< 0.05) were included in the multivariable logistic regression analysis. In multivariable logistic regression analysis, the variables of age(OR,1.038; 95% CI, 1.006–1.071; *P *= 0.018), left atrial diameter (LA)(OR,1.069; 95% CI, 1.007–1.135; *P* = 0.029), right atrial diameter (RA)(OR,1.064; 95% CI, 1.007–1.125; *P* = 0.027), preoperative red blood cells (RBC) (OR,0.438; 95% CI, 0.230–0.833; *P* = 0.012), previous acute coronary syndrome (preACS) (OR,7.658; 95% CI, 2.949–19.885; *P* < 0.001) were independently associated with new-onset POAF (Table 3). The collinearity diagnostic analysis showed that the VIFs of those risk factors were less than 5, suggesting that there is no strong indication of multicollinearity among variables. Thus, five variables were included in the final multivariable prediction model as predictors.

**Table 2:** Univariable logistic regression analysis showing the risk variables of new-onset POAF after Sun's surgery.

Variable	OR	95% CI	*P* valve
Age (years)	1.044	1.015–1.074	0.002
LA (mm)	1.066	1.011–1.124	0.018
RA (mm)	1.066	1.016–1.121	0.010
WBC (×10^9^ /L)	0.905	0.772–0.966	0.068
RBC (×10^9^ /L)	0.447	0.246–0.760	0.004
FIB (g/L)	0.787	0.609–1.017	0.067
preACS	5.956	2.545–13.938	0.000

OR, odds ratio; CI, confidence interval; LA, left atrial diameter; RA, right atrial diameter; WBC, preoperative white blood cells; RBC, preoperative red blood cells; FIB, preoperative fibrinogen; preACS, previous acute coronary syndrome.

**Table 3:** Multivariable logistic regression analysis showing the risk variables of new-onset POAF after Sun's surgery.

Variable	Odds ratio (95% CI)	*P* value
Age (years)	1.038 (1.006–1.071)	0.018
LA (mm)	1.069 (1.007–1.135)	0.029
RA (mm)	1.064 (1.007–1.125)	0.027
RBC (×10^9 ^/L)	0.438 (0.230–0.833)	0.012
preACS	7.658 (2.949–19.885)	0.000

CI, confidence interval; LA, left atrial diameter; RA, right atrial diameter; RBC, preoperative red blood cells; preACS, previous acute coronary syndrome.

### Nomograms and model performance

**Figure 2:**
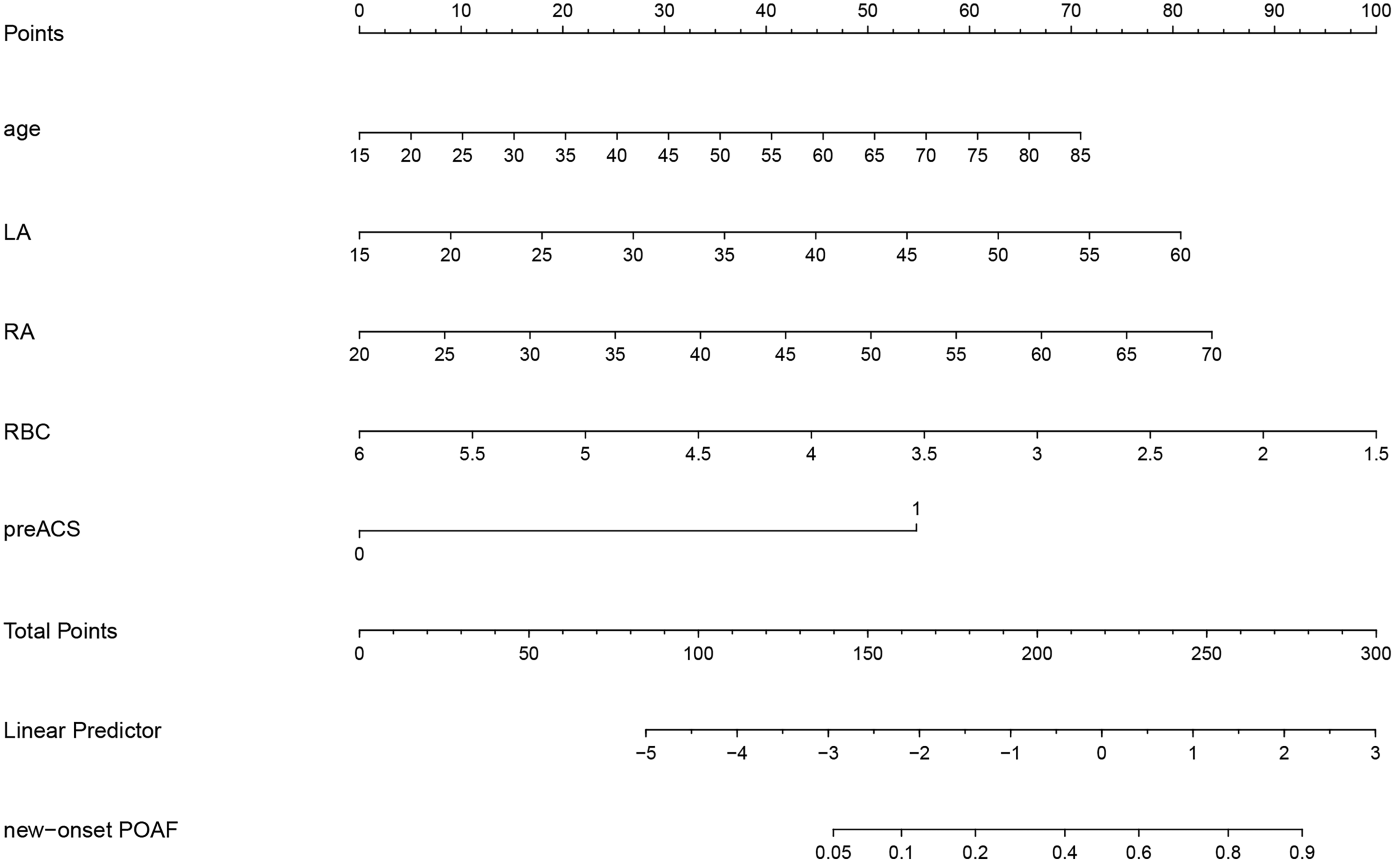
Nomogram prediction of new-onset POAF after Sun's surgery. The steps are: Determine the value of the variable on the corresponding axis, draw a vertical line to the total points axis to determine the points, add the points of each variable, and draw a line from the total point axis to determine the new-onset POAF probabilities at the lower line of the nomogram.

**Figure 3:**
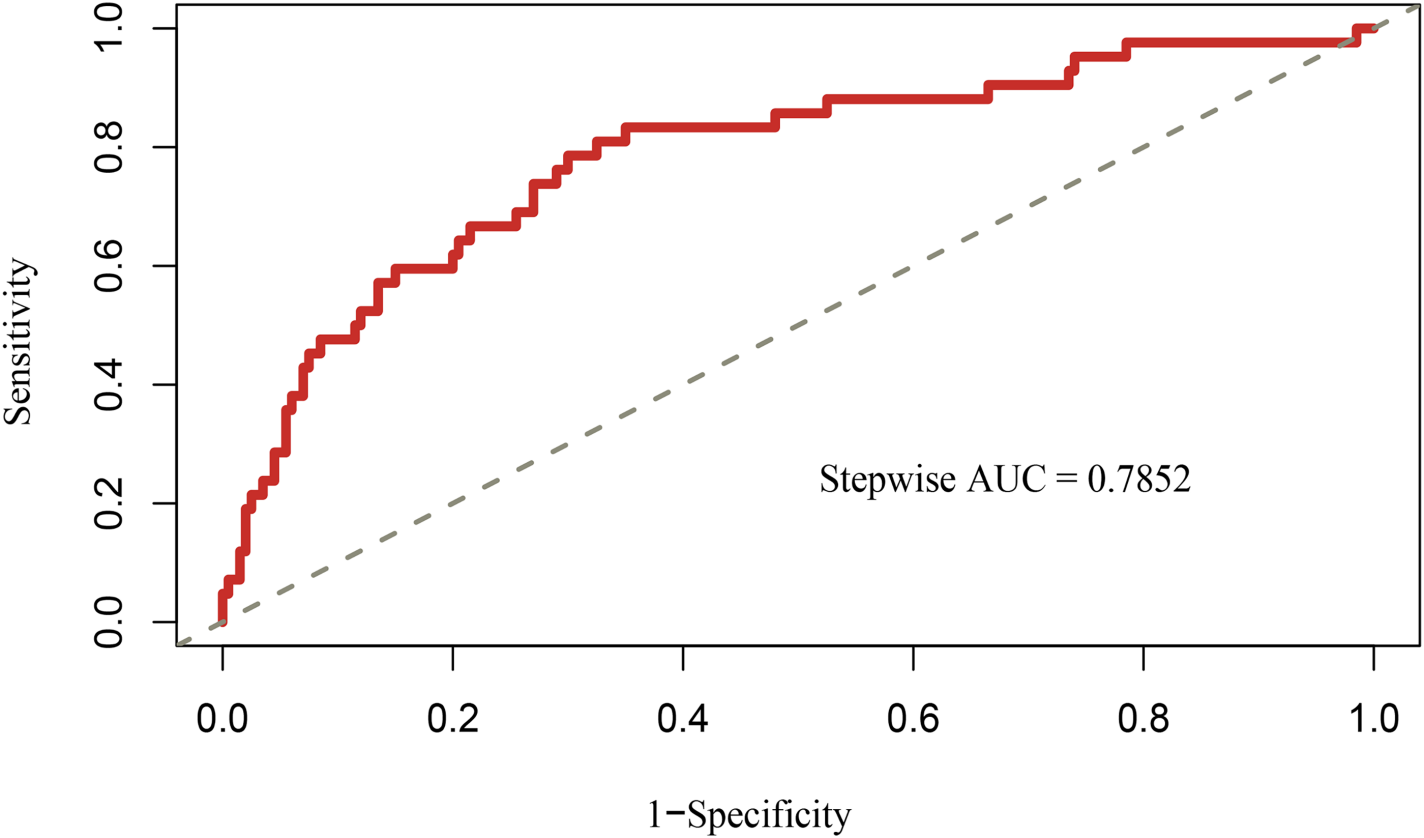
Receiver operating characteristic curves of nomogram for predicting new-onset POAF after Sun's surgery. AUC, area under the receiver operating characteristic curve.

**Figure 4:**
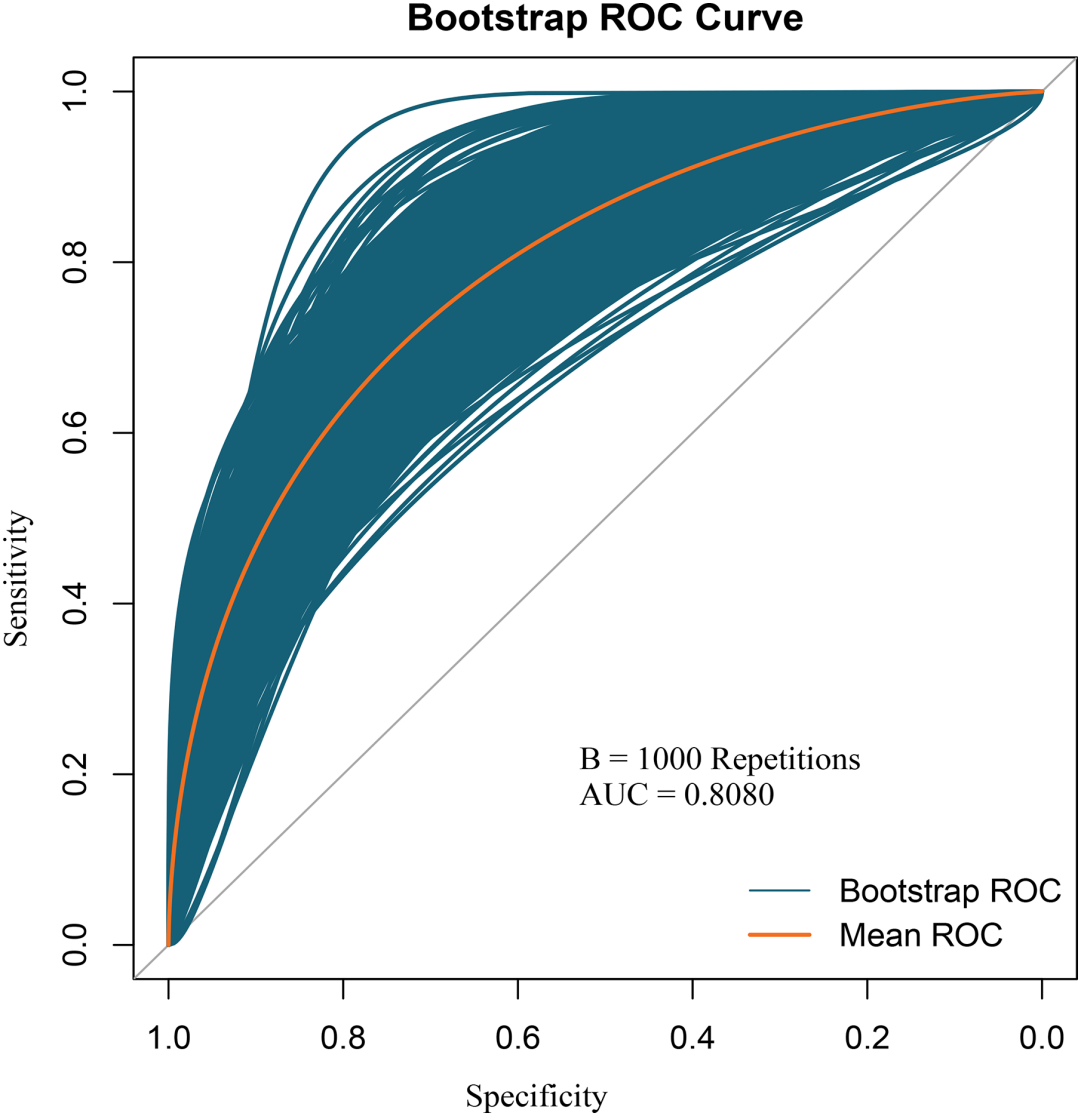
Internal validation of the nomogram using the bootstrap sampling. The ROC curve was measured by bootstrapping for 1,000 repetitions, and the AUC of the bootstrap stepwise model was showed.

**Figure 5:**
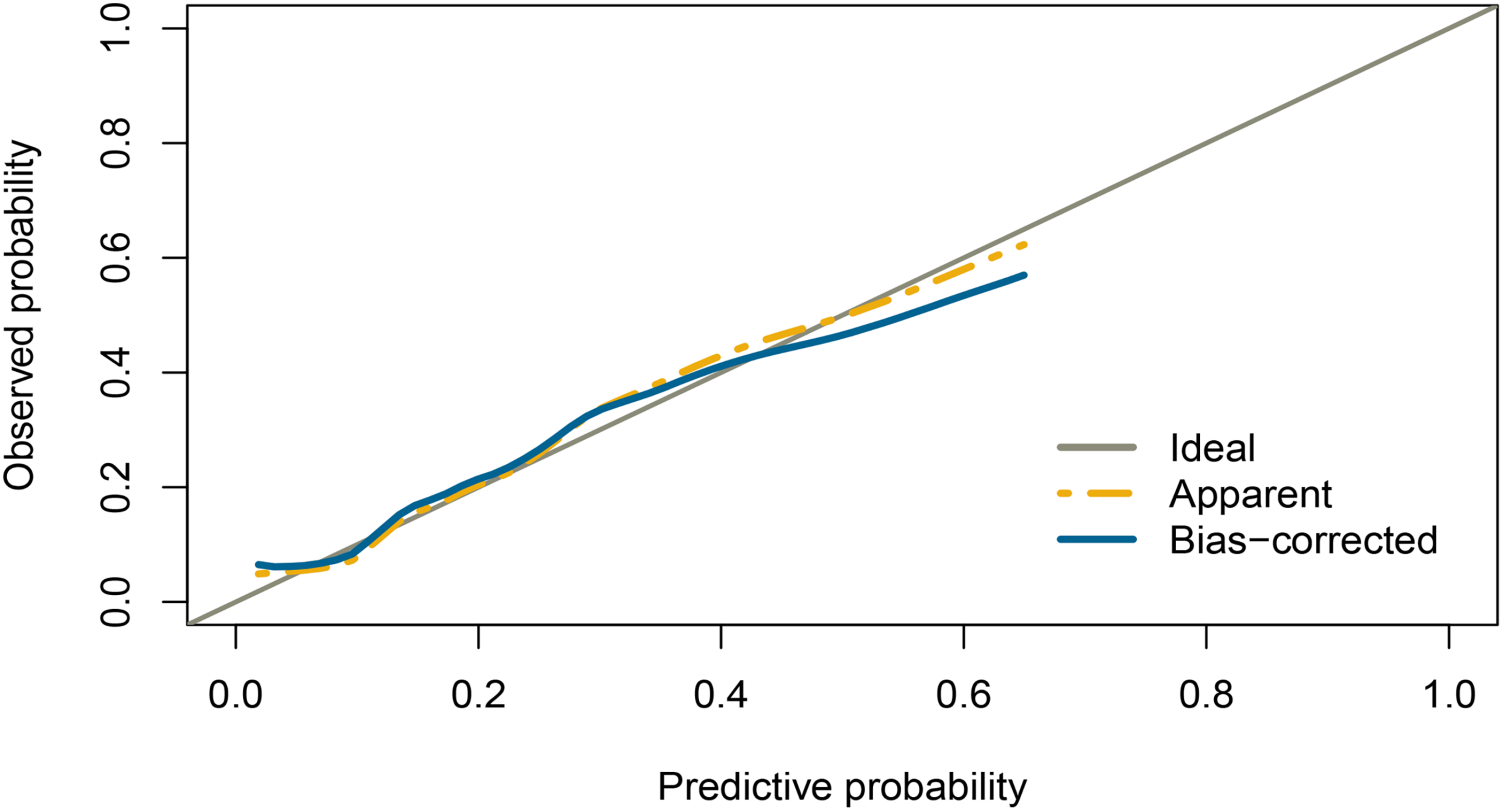
Internal validation of the nomogram using the bootstrap sampling. Calibration curve for predicted probability of the new-onset POAF nomogram. The curves describe the calibration of the nomogram in terms of agreement between predicted risks (x-axes) and actual outcomes (y-axes). The diagonal line indicates perfect prediction by an ideal model. The curve indicates the performance of the model.

**Figure 6:**
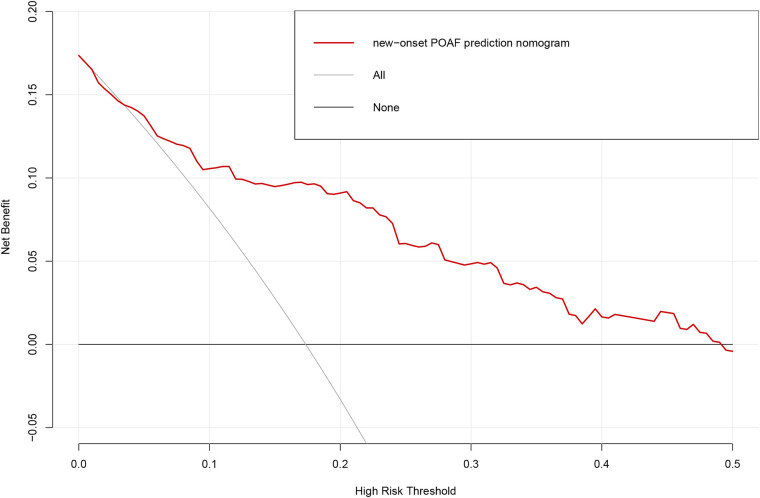
Decision curve analysis for the prediction model. Red solid line: Prediction model. Tin slash line: Assume all patients have new-onset POAF. Solid horizontal line:Assume no patients have new-onset POAF. The graph indicates the expected net benefit per patient relative to the nomogram prediction of new-onset POAF. POAF: Postoperative atrial fibrillation.

Based on the multivariable logistic regression analysis, a nomogram was developed to predict new-onset POAF after Sun's surgery for AAAD, incorporating 5 significant risk factors: age, LA, RA, RBC, and preACS (Figure 2). The final score, obtained by combining the individual scores, was used to assess the probability of new-onset POAF. The performance of this nomogram was measured using ROC curve analysis, and the area under the ROC curve (AUC) of this model was 0.7852, indicating the prediction model's accuracy and reasonable discriminative ability (Figure 3). The stepwise nomogram underwent internal bootstrap validation enhence its robustness. Utilizing bootstrapping with 1,000 repetitions, the ROC curve was generated, yielding an AUC of 0.8080 (95% CI: 0.8056–0.8104), indicative of comparable statistical power to the initial stepwise model (Figure 4). The nomogram's predictive performance was further validated through bootstrap self-sampling and internal validation procedures conducted 1,000 times, generating a calibration curve that confirms a satisfactory fit (Figure 5) and the HL test showed that our predicted and observed values were close (*P* = 0.272). Furthermore, decision curve analysis illustrated the favorable potential clinical effect of the predictive model (Figure 6).

## Discussion

AAAD represents a grave medical condition necessitating a well-coordinated multidisciplinary approach for prompt diagnosis and intervention. Despite advancement in immediate surgical intervention substantially enhancing survival rates following AAAD, the operative mortality remains considerable (20). Recent studies have reported an in-hospital mortality rate of 22% (21, 22). However, with the rise in AAAD surgical procedures and the further refinement of a variety of new surgical techniques and adjuctive technologies, postoperative mortality rate from AAAD surgery has been significantly reduced. Currently, the survival rate for AAAD at specialized surgical centers stands at 85%–90% (23). Nonetheless, the incidence of POAF, one of the common complications after AAAD surgery, has not witnessed a proportionate improvement (8, 9). A meta-analysis comprising 32 studies and 155,575 patients revealed a 23.7% occurrence of POAF following cardiac surgery (15). In addition, according to the limited literature, the incidence of POAF after aortic arch repair ranges from 19.6% to 57.2% (2, 24, 25). In our research, the incidence of AF in patients with AAAD who underwent Sun's surgery was 17.36%, notably lower than previously documneted rates. This disparity may be attributed to the following reasons: (1) Our stringent definition of POAF, in alignment with the criteria outlined by the Society of Thoracic Surgeons National database, which entails the presence of AF persisting for five minutes or longer on at least two consecutive days of electrocardiogram monitoring, or the manifestation of atrial flutter requiring medical intervention. This definition diverges from certain prior definitions found in literature, which may include criteria like AF exceeding 10 min or any occurrence of AF. (2) We studied predominantly new-onset POAF, resulting in exclusion criteria differing from previous studies in that we excluded patients with a prior history of AF. (3) The demographic characteristics of our study population differed, with our participants being notably younger, compared to participants in previous investigations (52.7 years vs. 68.7 years) (2). Additionally, a majority of our patients did not present with concomitant cardiac dysfunction, potentially influencing the observed incidence rates.

Evidence indicates that patients with AF prior to surgery are more predisposed to persistence of AF during both the immediate and protracted stages of postoperative recovery, while those without preoperative AF exhibit significantly reduced susceptibility to develop POAF. Undoubtedly, preoperative AF stands out as a significant risk factor for postoperative AF (2, 26). In this study, to specifically study new-onset AF after surgery, patients with previous AF were excluded. Although POAF is a transient, self-limiting postoperative complication and is not life-threatening, there is a risk of hemodynamic impairment and thromboembolism (27). Moreover, it may lead to prolonged hospital stay and higher cost (2). Prior research has demonstrated a strong association between POAF following aortic surgery and extended hospitalization, postoperative liver dysfunction, and in-hospital mortality (25, 28). In addition, patients who developed POAF had a 5-fold increased risk of developing permanent AF (29). A prompt diagnosis to identify those patients with a high chance of POAF after Sun's surgery in the hospital would help manage the illness and improve patient's outcomes. In spite of this, there are currently no predictive models to assess the risk of POAF in patients who have had Sun's surgery. In this research, we developed and validated a new and straightforward nomogram for recognizing high-risk patients, thereby assisting clinicians in devising more effective treatment. The indicators included in this study are all routine cardiovascular surgical examination items, following the principle of facilitating clinical dissemination.

Although risk factors for POAF after AAAD surgery have been identified in previous studies, comprehensive analysis within AAAD populations remains deficient. The findings of this study revealed that age, LA, RA, RBC, and preACS independently influenced the occurrence of new-onset POAF in patients with AAAD (*P* < 0.05). In our nomogram, preACS emerged as the most significant factor associated with an increased risk of new-onset POAF.

Numerous studies have substantiated that age constitutes a risk factor for the onset of AF following cardiac surgery in patients (2, 15, 30). Elderly patients with advanced age typically exhibited hightened prevalence of chronic diseases, as alongside degenerative alterations in cardiovascular structure and electrophysiological abnormalities. Clinical epidemiological studies have consistently demonstrated an increasing risk of AF as age advances (31–33). The correlation between age and POAF incidence exhibits a non-linear pattern. Individuals aged over 55 demonstrates a markedly elevated POAF rate in contrast to their counterparts below 55. Moreover, with each decade of age, the incidence of POAF approximately doubles (18). Our study cohort indeed encompasses a younger age range compared to participants in previous investigations, which may also be a reason for our low incidence of POAF. To ensure that our findings are applicable to a broader patient population, we should expand our sample size to include a wider age range of patients in future studies.

PreACS is an additional risk factor that may elevate the occurrence of new-onset POAF in patients with AAAD. Recent research has indicated that myocardial infarction can be a cause of AF (34). In a study by Muayad Alasady et al., acute myocardial infarction was found to predisposed individuals to AF when affecting the gyratory and atrial branches of the coronary arteries (35). The mechanism may involve two factors: (1) Marked reduction in coronary blood flow to the atria impairs atrial myocyte excitability and conduction velocity, thereby triggering the refractory mechanism and AF. (2) The autonomic nervous system, including the sympathetic and parasympathetic branches, also significantly contributes to the development of AF (36).

Multiple studies have provided evidence indicating that preoperative echocardiography parameters related to the left atrium, such as increased left atrial diameter or left atrial enlargement, are linked to the subsequent development of POAF (37–39). Atrial enlargement is frequently attributed to changes in cardiac hemodynamics, elevated atrial and ventricular pressures, suggesting potential impairment of cardiac function. Moreover, atrial enlargement triggers structural remodeling and modifications in the electrophysiological properties of myocardial cells, both playing a significant role in the initiation and perpetuation of AF (40).

In this study, we identified the preoperative red blood cell (RBC) count as an independent protective factor against AF in patients with AAAD who underwent Sun's surgery. While the precise relationship between preoperative red blood cell counts and AF following cardiac surgery remains to be fully elucidated, Imataka and colleagues discovered a significant impairment in plasma volume and erythrocyte biology among patients with AF in their study (41). Consequently, it is proposed that a pre-operative low red blood cell count could be a potential hazard for new-onset POAF following Sun's surgery. Future researchers may be able to validate this hypothesis with prospective studies.

Numerous accounts highlight various factors related to the development of POAF following cardiovascular surgery. There is substantial evidence indicating that inflammation plays an important role in the etiology of POAF. Many investigations have indicated that inflammation can modify atrial conduction, thereby increasing the risk of POAF developmen (42, 43). Some studies have reported that, elevated NLR in the context of cardiovascular disease is associated with POAF (44, 45). It has been proposed that heightened inflammatory biomarker in AF could be an indication of atrial inflammation, leading to electrical and structural remodeling of the atria, thus initiating AF. Besides, inflammation can upset calcium balance and instigate diverse atrial conduction, which could increase the vulnerability to AF (46). However, in this study, no significant impact of pertinent inflammatory markers on the occurrence of new-onset POAF was observed. Perzanowski and his colleagues found that removing the aortic fat pad could alter the autonomic nerve supply to the heart, potentially elevating the likelihood of AF (24). However, our study, focused on routine preoperative checkups, did not specifically investigated this aspect. Higher body mass index has been associated with a greater occurrence of POAF (8). Obesity, characterized by increased cardiac output demand, and larger left ventricular and left atrial sizes is linked to an increased likelihood of developing POAF (47). However, our study did not find any significant association between new-onset POAF and obesity, which may be due to the small sample size. Huang and colleagues elucidated the impact of atrial dilation on electrophysiological characteristics and the inducibility of AF (48). The extent of atrial dilatation is contingent upon intraoperative and postoperative fluid balance. Thus, it is hypothesized that fluid volume management is associated with the occurrence of POAF, warranting further investigation.

The survival rate of patients with AAAD diminishes progressively during the acute phase, and there is a consensus that surgical intervention should be expedited. In developed countries, the mean interval duration from arrival at the emergency department to diagnosis and from diagnosis to surgery of AAAD patients was 4.3 h (IQR = 22.5 h) and 4.3 h (IQR = 21.6 h), respectively (49). In contrast, the average interval from symptom onset to surgical intervention in China was 5.0 ± 3.8 days, which was attributed to disparities in healthcare resource distribution and greater transfer distances (5). As the duration of the dissection increases, it exposes the extracellular matrix to inflammatory cells in the bloodstream, exacerbating the systemic inflammatory response. The progression of the dissection and the development of the inflammatory response mutually reinforce each other, worsening the condition concurrently. A significant number of patients with AAAD also experience systemic inflammatory response syndrome (SIRS). And many investigations have indicated that inflammation can alter atrial conduction, thereby increasing the risk of POAF development (42, 43). Therefore, it is hypothesised that the time between symptom onset and surgical intervention in patients with AAAD is associated with the occurrence of new-onset POAF and warrants further investigation.

In our series, most newly developed AF cases were transient. We adhere to a standardized protocol of administering *β*-blockers or amiodarone therapy when there were no contraindications. Only a small number of patients persisted with AF symptoms at discharge. For these patients, anticoagulation therapy was initiated to mitigate the risk of thromboembolic events. Patient management, especially for elderly individuals with atrial enlargement and preACS, necessitates closer monitoring of cardiac rhythm and electrolyte status, alongside prompt intervention upon detection of POAF. In case of anemia preoperatively, red blood cell transfusion is actively employed for correction. Furthermore, intraoperative surgical techniques ae meticulously optimized to minimize operative duration and mitigate red blood cell loss, thereby potentially reducing the likelihood POAF.

### Limitations

The present study is subject to several limitations. Firstly, our sample pool originated exclusively from a single institution, which may limit the generalizability of our findings. Thus external validation of the proposed nomogram in diverse cohorts is essential in subsequent studies. Secondly, the prediction model was retrospectively constructed, introducing inherent biases that necessitate validation through prospective studies to establish its reliability and clinical applicability. Lastly, while efforts were made to optimize the accuracy of the nomogram, its performance may still fall short of achieving a high level of reliability, potentially leading to misdiagnosis in critical clinical scenarios. Therefore, ongoing refinement and validation of the nomogram are imperative to enhance its clinical utility and accuracy.

## Conclusions

In summary, we found that age, LA, RA, RBC, and preACS were independent influencing factors on the occurrence of new-onset POAF in AAAD patients. Based on these factors, a nomogram was designed to predict the risk of new-onset POAF in patients with AAAD. The results suggest that this predictive model may be effective. Utilizing the nomogram allows for individual predictions for each patient, optimizing treatment plans specifically for those with new-onset POAF arising from Sun's surgery. In elderly patients with atrial enlargement and preACS, there is a critical need for heightened postoperative monitoring of cardiac rhythm and electrolyte balance, with swift intervention for new-onset POAF.

## Data Availability

The raw data supporting the conclusions of this article will be made available by the authors, without undue reservation.
